# A New Oviraptorid Dinosaur (Dinosauria: Oviraptorosauria) from the Late Cretaceous of Southern China and Its Paleobiogeographical Implications

**DOI:** 10.1038/srep11490

**Published:** 2015-07-02

**Authors:** Junchang Lü, Hanyong Pu, Yoshitsugu Kobayashi, Li Xu, Huali Chang, Yuhua Shang, Di Liu, Yuong-Nam Lee, Martin Kundrát, Caizhi Shen

**Affiliations:** 1 Institute of Geology, Chinese Academy of Geological Sciences, Beijing 100037, China; Key Lab of Stratigraphy and Paleontology, Ministry of Land and Resources of China, Beijing 100037, China; 2 Henan Geological Museum, Zhengzhou 450016, Henan, China; 3 Hokkaido University Museum, Hokkaido University, Sapporo, Japan; 4 Korea Institute of Geoscience and Mineral Resources, Daejeon, South Korea; 5 Department of Organismal Biology, Evolutionary Biology Centre, Uppsala University, Uppsala, Sweden

## Abstract

The Ganzhou area of Jiangxi Province, southern China is becoming one of the most productive oviraptorosaurian localities in the world. A new oviraptorid dinosaur was unearthed from the uppermost Upper Cretaceous Nanxiong Formation of Ganzhou area. It is characterized by an anterodorsally sloping occiput and quadrate (a feature shared with *Citipati*), a circular supratemporal fenestra that is much smaller than the lower temporal fenestra, and a dentary in which the dorsal margin above the external mandibular fenestra is strongly concave ventrally. The position of the anteroventral corner of the external naris in relation to the posterodorsal corner of the antorbital fenestra provides new insight into the craniofacial evolution of oviraptorosaurid dinosaurs. A phylogenetic analysis recovers the new taxon as closely related to the Mongolian *Citipati*. Six oviraptorid dinosaurs from the Nanxiong Formation (Ganzhou and Nanxiong) are distributed within three clades of the family. Each of the three clades from the Nanxiong Formation has close relatives in Inner Mongolia and Mongolia, and in both places each clade may have had a specific diet or occupied a different ecological niche. Oviraptorid dinosaurs were geographically widespread across Asia in the latest Cretaceous and were an important component of terrestrial ecosystems during this time.

Oviraptorosaurs are an unusual group of feathered dinosaurs, which are easily distinguished from other maniraptoran theropods by their unique skull and ischial morphologies. Basal forms such as *Caudipteryx*^1^ have teeth, whereas derived members of the clade are united by synapomorphies such as loss of the teeth, short skulls, variable presence of the bony crest, and pneumatic anterior caudal vertebrae^2^. Skeletally mature oviraptorosaurs ranged in size from the turkey-sized *Caudipteryx*^1^ to the 8-meter long *Gigantoraptor*^3^. Since the first discovery of *Oviraptor* in 1924^4^, more than thirty genera have been reported. They are mainly from the Cretaceous of Asia^3^,^4^,^5^,^6^,^7^,^8^,^9^,^10^,^11^,^12^,^13^,^14^,^15^,^16^,^17^,^18^,^19^,^20^,^21^,^22^,^23^,^24^,^25^,^26^,^27^,^28^,^29^ and North America^30^,^31^,^32^,^33^,^34^,^35^,^36^,^37^,^38^,^39^. The Chinese oviraptorosaurs are mostly from the Lower Cretaceous deposits of western Liaoning Province and the Upper Cretaceous deposits of Inner Mongolia (northern China), the upper Lower Cretaceous of the Ruyang Basin and uppermost Cretaceous of the Tantou Basin in Henan Province (central China), and the Upper Cretaceous deposits of the Heyuan and Nanxiong basins of Guangdong Province and the Ganzhou district of Jiangxi Province (southern China). Until now, four genera of oviraptorosaurs have been reported from the Upper Cretaceous Nanxiong Formation of Ganzhou City, Jiangxi Province of southern China: *Banji*^21^, *Ganzhousaurus*^28^, *Jiangxisaurus*^29^ and *Nankangia*^26^. Within an area of about 40 square kilometers, more than 200 oviraptorosaurian nests with eggs have been discovered in the Ganzhou region^40^. They did not come from a single quarry but they are mainly from Longling village of Nankang City, and sites near the Ganzhou Railway Station, and the third high school of Ganxian, Ganzhou City; some nests contain eggs with embryos inside them^41^. In addition to remains of the oviraptorosaur skeletons, lizards and other dinosaurs, including the large sauropod *Gannansaurus sinensis*^42^ and the tyrannosaurid *Qianzhousaurus sinensis*^43^, have also been excavated from the Nanxiong Formation of the Ganzhou area. A new oviraptorid taxon was unearthed from the current construction site of the Ganzhou Railway Station (Fig. 1). The new specimen reported herein is from the Upper Cretaceous Nanxiong Formation of Ganzhou, Jiangxi Province. It is distinctive from any other reported oviraptorosaurs from southern China, but does show some similarities to the Mongolian oviraptorid *Citipati*.

## Results

Systematic Paleontology

Oviraptorosauria Barsbold, 1976^5^.

Oviraptoridae Barsbold, 1976^5^.

Oviraptorinae Barsbold, 1981^44^.

**Figure 2:**
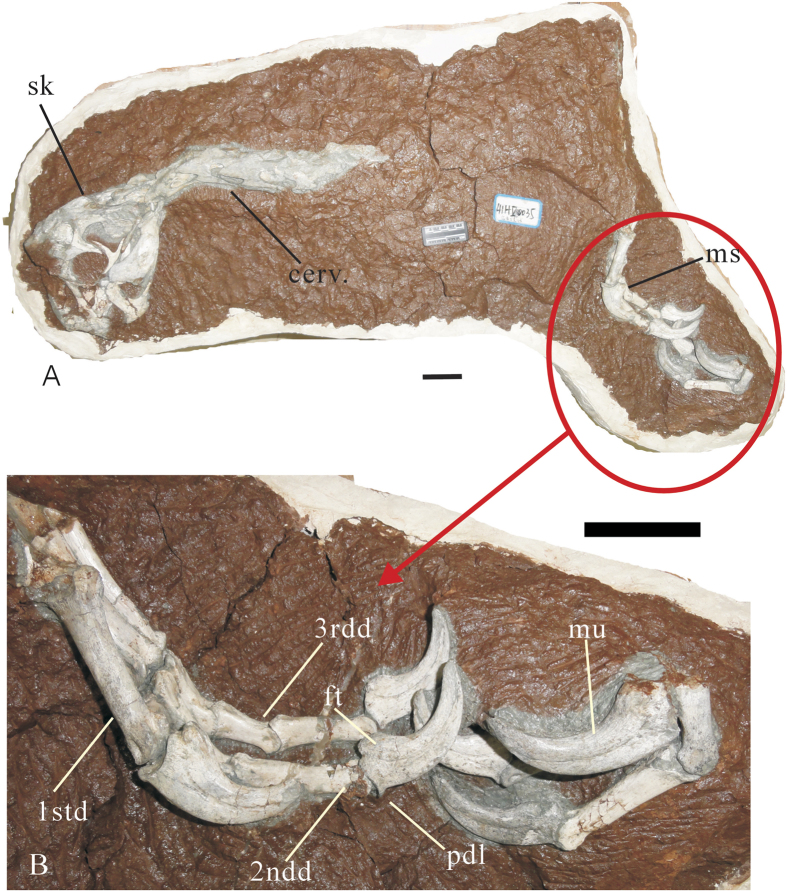
The holotype of *Huanansaurus ganzhouensis* (HGM41HIII-0443) gen. et sp. nov. (**A**); a close up of the phalanges of the right and left hand (**B**). Abbreviations: 1std., the first digit; 2ndd., the second digit; 3rdd., the third digit; cerv., cervical vertebrae; ft., flexor tubercle; ms., manus; mu., manual ungual; pdl., proximodorsal lip; sk., skull. Scale bar = 5 cm.

**Figure 3:**
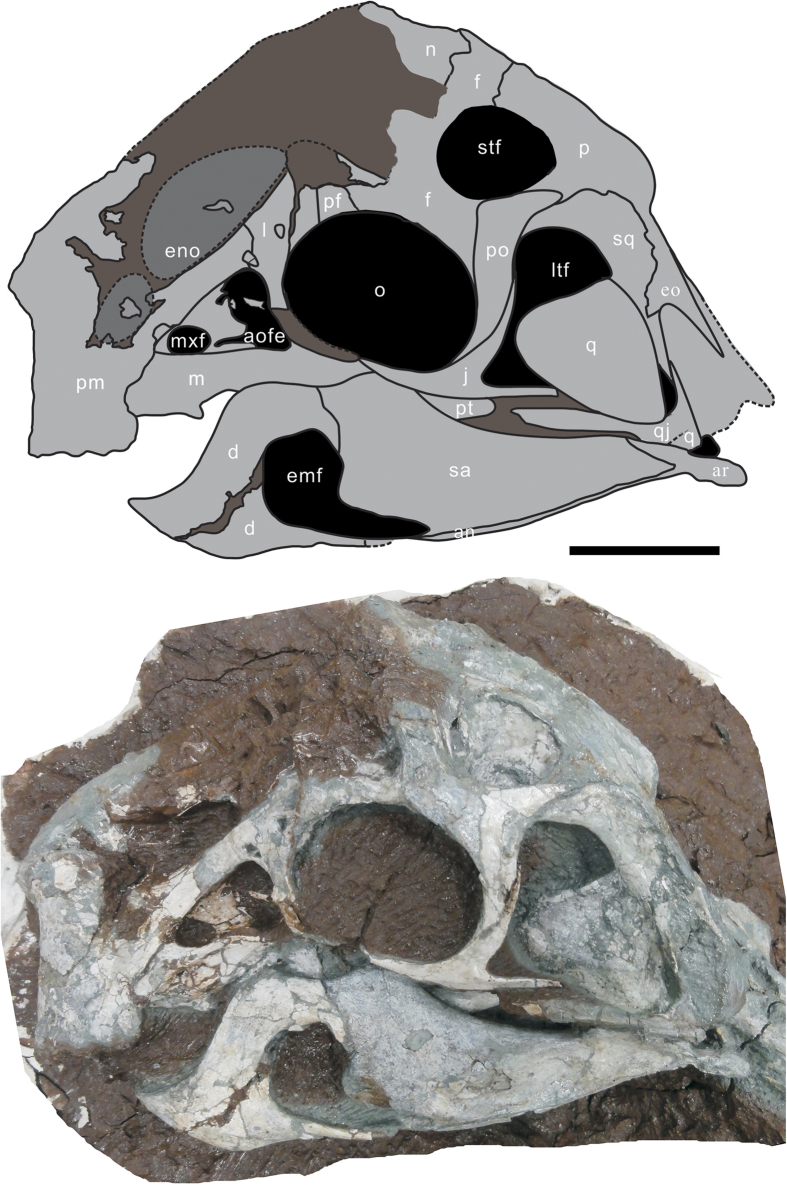
Outline (**A**) and photograph (**B**) of the skull of *Huanansaurus ganzhouensis* (HGM41HIII-0443) gen. et sp. nov. Abbreviations: an., angular; aofe., antorbital fenestra; ar., articular; d., dentary; emf., external mandibular fenestra; eno., external narial opening; eo., exoccipital; f., frontal; j., jugal; l., lacrimal; ltf., lower temporal fenestra; m., maxilla; mxf., maxillary fenestra; n., nasal; p., parietal; pf., prefrontal; pm., premaxilla, po., post orbital; pt., pterygoid; q., quadrate; qj., quadratojugal; sa., surangular; sq., squamosal; stf., supratemporal fenestra. Scale bar = 5 cm.

*Huanansaurus ganzhouensis* gen. et sp. nov. (Figs 2,3)

### Etymology

Generic name refers to “Huanan” (in Chinese Pinyin), which means southern China, because the dinosaur was discovered in Ganzhou of Jiangxi Province. The specific name refers to the locality of Ganzhou.

### Holotype

Partial skeleton with a nearly complete skull (HGM41HIII-0443); accessioned at the Henan Geological Museum, Zhengzhou, China.

### Type locality and horizon

In the vicinity of the Ganzhou Railway Station (GPS coordinates are provided on request from the first author), Ganzhou City; Campanian-Maastrichtian; Nanxiong Formation (Upper Cretaceous)^45^.

### Diagnosis

A new oviraptorid dinosaur bears the following unique combination of characters, with several autapomorphies indicated with an asterisk: mandibular condyles of quadrate are posterior to the occipital condyle*; nuchal transverse crest is pronounced*; angular contributes extensively to the border of the external mandibular fenestra*; dentary, anterodorsal tip of beak projecting anterodorsally, with tip of beak projecting at an angle of 45° or less relative to the ventral margin of the symphysis*; pneumatized dentaries*; metacarpal I is long and slender, diameter 20% of length*; posteroventral branch of dentary twisted so that lateral surface of branch faces somewhat ventrally*; development of symphyseal shelf of mandible is intermediate, length of symphysis greater than 20% but less than 25% when compared with the mandible*; proximodorsal extensor ‘lip’ on each manual ungual is prominent (‘set off’ from remainder of dorsal surface by distinct change in slope immediately distal to ‘lip’) *; the circular supratemporal fenestra much smaller than the lower temporal fenestra; the posterodorsal process of the premaxillae contact the lacrimals and there is a distinct opening near the posteroventral corner of its distal end; dorsal margin of dentary above the external mandibular fenestra where it is strongly concave ventrally. *Huanansaurus* differs from most oviraptorosaurs by the anterodorsally sloping occiput and quadrate (as in *Citipati*), and the anteroventral corner of the external naris is slightly below the horizontal line projected through the posterodorsal corner of the antorbital fenestra (character shared with *Citipati osmolskae* [MPC-D 100/978], *Citipati* sp. [MPC-D 100/42], *Khaan*, and *Yulong mini*); posterior margin of the fourth cervical forms straight line between the postzygapophyses in dorsal view (shared with *Yulong*).

Although there is only a single overlapping element (the proximal end of tibia) between *Huanansaurus* and *Shixinggia*, *Huanansaurus* clearly differs from *Shixinggia*^18^ in that there is a distinct opening on the medial surface near the proximal end of the tibia in *Shixinggia*, whilst this opening does not appear in the *Huanansaurus*.

*Huanansaurus* differs from *Heyuannia*^13^,^14^ in that the symphyseal portion of the dentary is strongly downturned, pneumatic foramina are present on the neural arches and ribs of cervical vertebrae, and the metacarpal III is strongly reduced in *Heyuannia*, whereas the symphyseal portion of the dentary is not downturned, there are no pneumatic foramina on the neural arches and ribs of cervical vertebrae, and the metacarpal III is in normal size in *Huanansaurus*.

*Huanansaurus* differs from *Ganzhousaurus*, *Jiangxisaurus* and *Nankangia* in that the dorsal margin of the anterior part of the dentary is strongly concave, whereas it is nearly straight in *Ganzhousaurus*^28^, *Jiangxisaurus*^29^ and *Nankangia*^26^.

*Huanansaurus* differs from *Wulatelong*^27^ in that the manual unguals have distinct lips on their proximodorsal ends, however, there is no lip at the proximodorsal ends of manual unguals, the anteroventral corner of the external narial opening below the horizontal line projected through the posterodorsal corner of the antorbital fenestra and size of metatarsal III is reduced in *Wulatelong*^27^.

*Huanansaurus* differs from *Banji*^21^ in that the external nasal opening of *Huanansaurus* is relatively smaller than that of *Banji*, and the lower temporal fenestra is trapezoidal in contrast with *Banji*, in which it is nearly square. The anteroventral corner of the external narial opening is far below the level of the posterodorsal corner of the antorbital concavity in *Banji*.

**Figure 4:**
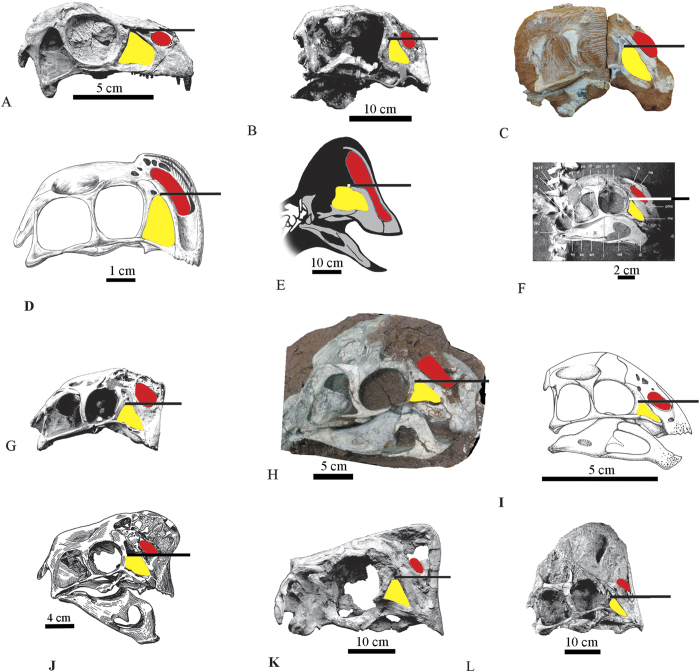
Skull comparisons of oviraptorosaurs showing relative positions of the posterodorsal corner of the antorbital fenestra and the anteroventral corner of the external narial opening. **A**: *Incisivosaurus gauthieri*; **B**: *Conchoraptor gracilis*; **C**: *Wulatelong gobiensis*; (no scale) **D**: *Banji long*; **E**: *Anzu wyliei*; **F**: *Khaan mckennai*; **G**: *Citipati osmolskae*; (no scale) **H**: *Huanansaurus ganzhouensis* (reversed); **I**: *Yulong mini*; **J**: *Oviraptor philoceratops*; **K**: *Nemegtomaia barsboldi*; **L**: “*Oviraptor*” *mongoliensis*. **A**, **B**, **F**, **G**, **J**, **K**, and L are from Lü^14^; C is modified from Xu *et al.*^27^; D is modified from Xu and Han^21^; E is modified from Lamanna *et al.*^39^ (reversed) and I is from Lü *et al.*^25^. External narial opening is in red, and antorbital fenenstra is in yellow. Note: The horizontal line projected through the posterodorsal corner of the antorbital fenestra is parallel to the line linking the articular end of the quadrate and the ventral margin of the premaxillae.

*Huanansaurus* differs from *Citipati*^11^ in that the parietal is much shorter along the midline than the frontal, the ascending process of the jugal extends posterodorsally, and the ventral margin of the posterodorsal process of the dentary above the external mandibular fenestra is strongly concave. The upper margin of the lower temporal fenestra in *Citipati*^11^ is nearly equal to that of the ventral margin making the lower temporal fenestra nearly rectangular, whereas the lower temporal fenestra is nearly triangular in *Huanansaurus* (Fig. 3, Fig. 4).

*Huanansaurus* differs from *Nemegtomaia*^16^,^17^,^46^ in that the anteroventral corner of the external narial opening is above the level of the posterodorsal corner of the antorbital concavity, the supratemporal fenestra is circular, and the highest point of the skull is above the orbit Fig. 4).

*Huanansaurus* differs from *Rinchenia mongoliensis*^47^(= *Oviraptor mongoliensis*^48^; Osmólska *et al.*^15^) in that *Huanansaurus* lacks a convex crest on the frontals and parietals, the dorsal margin of the dentary above the external mandibular fenestra is concave, the anteroventral corner of the external naris is slightly below the horizontal line projected through the posterodorsal corner of the antorbital fenestra, whilst *Rinchenia mongoliensis* bears a distinct convex crest on the frontals and parietals, the anteroventral corner of the external naris is above the horizontal line projected through the posterodorsal corner of the antorbital fenestra in *Rinchenia* (Fig. 4).

*Huanansaurus* differs from *Khaan* in all unguals have distinct lips above the proximal articulations in *Huanansaurus,* and the posterior and ventral margins of the lower temporal fenestra form a nearly right angle in *Khaan*^48^ whereas the angle is acute in *Huanansaurus* (Fig. 4F, H).

### Description

The specimen has a nearly complete skull including lower jaws (Figs 2, 3; Supplementary Information). Seven cervical vertebrae are preserved; the first four are almost complete whereas the last three are only preserved as fragments or impressions. The partial humerus, ulna, radius and complete hand of the right arm, the left hand, a small portion of the distal end of the right femur, the proximal end of the right tibia, and the distal portion of the right pes are preserved.

The skull is highly pneumatic, especially the anterodorsal portion of the skull roof that includes the nasals, lacrimals and premaxillae.

The premaxillae are toothless and are fused. In lateral view, the anterior and ventral margins of the premaxilla form an angle of about 80 degrees (Fig. 3). The paired premaxillae are U-shaped in ventral view. The premaxilla bears both anterodorsal and posterodorsal dorsal processes. The posterodorsal process of the premaxillae is strap-like and there is a large opening near the posteroventral corner of its distal end. The posterodorsal process of the premaxilla extends to contact the lacrimals uniquely. In *Yulong*, the posterodorsal processes of the premaxillae contacts the nasals^25^, whereas they contact both lacrimals and nasals in *Citipati osmolskae*^11^and *Conchoraptor gracilis*^15^. The distal end of the anterodorsal process (nasal process) of the premaxilla along with the anterior portion of the nasal forms a distinct crest on the skull that is similar to that of the Zamyn Khondt (MPC-D100/42) oviraptorine, which has been referred to as *Citipati* n. sp.^15^. The ventral process of the premaxilla forms the anteroventral portion of the external naris. Anteroventral to the external nasal opening, there is a deeply concave surface on the premaxilla, similar to those of *Yulong*^25^ and *Nemegtomaia*^17^, but unlike that of *Citipati osmolskae*, where this area is slightly depressed^11^.The external narial opening is elongate, with its long axis extending anteroventrally/posterodorsally (Fig. 3).

As in other oviraptorid dinosaurs, the maxilla is edentulous and anteroposteriorly short in lateral view, and it is also excluded from the external naris. A distinct ridge on the lateral surface of the maxilla forms the boundary of the ventral margin of the antorbital fossa. There are two openings within the antorbital fossa; the anterior one (promaxillary fenestra) is suboval with a long axis extending anterodorsally/posteroventrally. It is smaller than the rectangular posterior one, which is closed posteriorly by the anterior margin of the lacrimal.

The nasals are missing, although their anterior portions are preserved as impressions. The estimated nasal length is longer than the combined lengths of the frontal and parietal. In lateral view, the nasal would have formed the upper border of the external narial opening. The anterior portions of the nasals are wedged between the posterodorsal processes of the premaxillae. Posteriorly, the nasal contacts the frontal at the highest region of the skull. The damaged surfaces of the nasals reveal numerous diverticula and thin bony struts. Such inner architecture indicates that the nasal bone is highly pneumatic. There is a small triangular bone at the anterodorsal corner of the orbit that may represent the prefrontal. It contacts the lacrimal anteriorly and the frontal posteriorly. There is an opening on the ventral surface of the lacrimal.

The lacrimal is composed of an anterior process that projects anteriorly, a preorbital process that extends ventrally, and main body that arches over the anterodorsal corner of the orbit. The anterior process of the lacrimal is wide and short. There is suture between this process and the posterodorsal process of the premaxilla. The main body (antorbital shaft) of the lacrimal is stout and forms the posterior border of the antorbital fenestra. There are three distinct openings on the lateral surface of the lacrimal. The anterior suboval opening (3.3 × 5.8 mm) is situated near the posterodorsal corner of the antorbital fossa. The middle opening is almost circular and has a diameter of 7.4 mm. The third, circular opening is posterodorsal to the middle opening, and has a diameter of about 4.5 mm. As in most theropod dinosaurs, the orbit is bound ventrally by the jugal and posteriorly by the postorbital (Fig. 3).

The frontals are fused with no sign of any suture. Central regions of the anterior parts of the frontals are only preserved as imprints. Sutures between the frontals and nasals can only be traced on imprints of the bones. There is a weak midline ridge that is formed by lateromedial depression on the dorsal surface of each frontal. Laterally, the frontal is overlapped by an anteromedial process of the postorbital, and together they rim the supratemporal fenestra anteromedially. A deep concavity on the dorsal surface of the anterolateral region of the frontal may indicate that the inside of the frontal is porous and has pneumatic diverticula as well

The parietals are fused and form the dorsal surface of 2/3 of the skull along the midline. The sutures with the frontals are nearly transverse. The parietals are rectangular in dorsal view. There is a weak ridge along the midline of the parietals. The posterior dorsal margin of the skull is not distinct, and the transverse nuchal crest is not pronounced. Unlike *Citipati*^11^, the occipital edge of the parietal is narrow.

The ventral margin of the jugal is ventrally convex (Fig. 3). The anterior part of the anterior process is missing. The broken surface shows that it was a thin plate-like bone, unlike the rod-like anterior process of the jugal in *Nemegtomaia*^16^,^17^. The jugal forms the entire ventral margin of the orbit. The posterior process of the jugal is short and thin, and is covered by the anterior process of the quadratojugal below the middle of the infratemporal fenestra. The postorbital process of the jugal extends posterodorsally, where it is covered by the ventral process of the postorbital. The jugal process of the postorbital is long, extending ventrally close to the base of the postorbital process of the jugal. The jugal forms 75% of the height of the postorbital bar. In lateral view, the postorbital and posterior (quadratojugal) processes form an acute angle of 70 degrees. A shallow, longitudinal groove on the ventral part of the posterior process is developed for the quadratojugal contact. The jugal forms most of the ventral and anterior margins of the large, trapezoidal infratemporal fenestra.

The quadratojugal has two distinct processes. The thin, plate-like anterior process and the ascending squamosal process meet at an angle of about 65 degrees. The straight anterior process is much longer than the ascending process. It differs from the rod-like anterior process in *Citipati osmolskae*^11^. The ascending process of the quadratojugal extends anterodorsally, and expands distally to contact the squamosal. The quadrate foramen is a large, suboval opening between the quadrate and the quadratojugal and is situated just dorsal to the posteroventral angle of the infratemporal fenestra. The quadratojugal is firmly fused with the quadrate, unlike the movable condition seen in *Nemegtomaia*^16^,^17^,^46^.

The quadrate is a massive bone among the elements of the skull. It is tightly sutured to the squamosal and quadratojugal, but loosely contacts the pterygoid. Similar to the quadrate of *Citipati*^11^, it is oriented obliquely in lateral view, extending posteroventrally from the squamosal. The distal articular surface forms two condyles that are separated by a longitudinal groove. The surface of the medial condyle is anteroposteriorly larger than that of the lateral condyle. The pterygoid flange of the quadrate is tall and extends posterodorsally to meet the short descending process of the squamosal as in derived oviraptorids.

The squamosal is a complex bone that forms the entire sharp dorsal margin of the infratemporal fenestra. Medially, its relationship with parietal is not clear. Anteriorly, it lies medial and ventral to the posterior process of the postorbital. It curves posterolaterally, and its distal end forks into a pair of tapering processes. The distal end of the quadrate is firmly fused with the squamosal. There is a small suboval foramen on the occipital surface between the squamosal and the paroccipital process. This opening is also present in *Citipati osmolskae*^11^.

The postorbital is a triradiate bone forming most of the postorbital bar. It bears three processes: the anterior, posterior and ventral processes. The anterior process is wide, short, and overlies the frontal anteriorly. The posterior process is relatively short and overlies the squamosal. The ventral process of the postorbital is long, tapered and is oriented nearly vertically. It almost reaches the posteroventral corner of the orbit. The shafts of the posterior and ventral processes of the postorbital meet at an angle of nearly 90 degrees. The postorbital forms the posterior border of a fossa situated dorsal to the orbit. The fossa extends into the pneumatic recesses of the narial region, as in *Citipati* os*molskae*^11^.

The supraoccipital is well-preserved. It is almost triangular in posterior view and has a distinct, vertical midline crest, similar to that of *Citipati osmolskae*^11^. In dorsal view, the supraoccipital is convex posteriorly. It is separated from the foramen magnum by the exoccipitals. The foramen magnum is nearly circular and much larger than the occipital condyle.

The paroccipital process is well preserved. It projects posterolaterally and ventrally from the foramen magnum. It is strongly pendulous, and extends below the level of the foramen magnum. There is a distinct ridge on the posterior surface of the paroccipital process that disappears before it reaches the sharply tapering distal end. There is a distinct concavity near the lateral edge of the foramen magnum on the posterior surface. The occipital condyle faces posteriorly; there is a shallow groove on its dorsal surface.

As in all derived oviraptorids, the edentulous mandible is dominated by a high, arching coronoid eminence. The dentary has two branches that rim the front of the large external mandibular fenestra. The ventral process is slender and extends posteriorly to contact the angular and surangular. The dorsal process is deep and extends posterodorsally to contact the surangular. The distance between the anterior margin of the external mandibular fenestra and the coronoid eminence is relatively long (4 cm/18.5 cm; the anteroposterior length of the external mandibular fenestra is 18.5 cm). The dorsal margin of the external mandibular fenestra is strongly concave ventrally, unlike those in any other oviraptorosaurs in which the margin is straight (*Anzu wyliei*^39^, *Citipati osmolskae*^11^, and *Incisivosaurus gauthieri*^12^), slightly convex (*Khaan mckennai*^49^) or slightly concave (*Caenagnathus collinsi*^31^,^33^, *Heyuannia*^13^ and *Nemegtomaia*^16^,^17^). The U-shaped mandibular symphysis is short and broad, with a transversely oriented, upturned anterior edge. The lateral surface of the mandibular symphysis is sculptured with irregularly distributed small pits. The dorsal margin of the mandibular symphysis is slightly below the level of the mandibular articulation. The dorsal surface of the symphysis is concave behind the anterior edge. The surangular and articular are fused in the posterior part of the lower jaw, with no clear suture between them. The coronoid eminence rises abruptly at the anterodorsal part of the surangular. The surangular descends gradually from the eminence and extends posteriorly to the end of the mandible, completely covering the lateral surface of the articular, as in *Citipati osmolskae*^11^ and *Nemegtomaia*^16^,^17^. The large mandibular fenestra is divided by an anterior process of the surangular; however, the anterior extent of the anterior process is unknown due to its incompleteness. The lateral surface of the surangular is shallowly depressed anterior to the mandibular articulation, but the central lateral surface is convex. There is a small opening anterior to this depression. Only small portion of the angular is exposed in lateral view along the ventral margin of the mandible.

The mandibular articulation is anteroposteriorly elongate and unbounded both anteriorly and posteriorly. The surface has a weak longitudinal midline ridge. The retroarticular process is long and descends posteroventrally from the articular surface. Its ventral edge descends slightly below the level of the remainder of the mandible. The mandibular articulation is much wider than the remainder of the mandibular ramus. Its relationships with prearticular and the surangular are not clear.

The atlas is in tight contact with the occipital condyle; its postzygapophyses are long. The prezygapophysis of the atlas is larger and much stronger than the postzygapophysis. The facet of the prezygapophysis is elongate with a slightly convex surface that faces anterodorsally. The facet is almost twice as large as the facet of the axial postzygapophysis, which may suggest that the neck was capable of extensive dorsoventral movement. The axis has an elongate pleurocoel; the neural spine is short and the postzygpophysis is relatively small and bears a weak epipophysis. The prezygapophysis of the axis extends farther anteriorly than the anterior margin of its centrum, but the postzygapophysis does not go beyond the posterior margin of the centrum. The postzygapophyses and epipophyses become progressively stronger from the third cervical vertebrae. In dorsal view, posterior margin forms a straight line between the postzygapophyses in each of the third and fourth cervical vertebrae, which is similar to those of *Yulong*^25^. The neural spine of the third cervical vertebra is missing. Its prezygapophysis extends anteriorly beyond the anterior margin of the centrum. The epipophysis is not developed. The cervical rib is long and is completely fused with the vertebra (Supplementary Information). The pleurocoel is larger than that of the axis and has a distinct anterior margin and open posterior margin. The ventral surface of the centrum is nearly flat. The anterior articular end is much wider than the posterior articular end.

The fourth cervical vertebra is complete. The neural spine is ridge-like. It is larger than the third cervical vertebra but their structures are similar to each other. The centrodiapophyseal lamina is stouter than that of the third cervical vertebra. The epipophysis is short and ridge-like, and is positioned anterior to the posterior margin of the postzygapophyses. Only the anterior portion of the fifth cervical vertebra is preserved.

The proximal portions of the right humerus are missing, but the length of the bone can be measured with the impressions of the missing parts. The shaft of the humerus is slightly twisted. The deltopectoral crest has a distinct ridge with a coarse surface along the medial margin of the posterior surface just below the level of the ventral margin. Near the distal end, the extensor surface of the shaft is gently depressed. The length ratio of radius to humerus is 0.97, much larger than those of *Khaan mckennai* (this ratio is 0.81)^49^ and *Jiangxisaurus ganzhouensis* (the ratio is 0.71)^29^.

The radius is nearly straight with expanded proximal and distal ends. The ulna is only preserved as an impression, but this provides an estimated length of the bone.

The right carpals, metacarpal I, and the proximal portions of metacarpals II and III are left as impressions. The first metacarpal is the shortest, and is less than half the length of metacarpal II, which is the longest metacarpal (Fig. 2B). Metacarpal III is 80% the thickness of metacarpal II. Metacarpal II is moderately robust, with the shaft diameter being about 13% of its length. The shaft is kidney-shaped in section, although the ventral surface of the shaft is nearly flat. The distal condyles are subcircular in lateral view. There is a wide, relatively deep groove on the ventral surface of the distal end, which appears to have permitted relatively extensive flexion–extension of the second digit. Prominent collateral ligament pits are present. The distal articular surface has a well-developed trochlea.

The shaft of metacarpal III is moderately robust, the diameter being about 11% of its length, unlike *Heyuannia* and *Jiangxisaurus*, where the metacarpal III is very weak and slender^14^,^29^. The shaft is straight and parallels the shaft of metacarpal II, and there is no space left between the two. In ventral view, the shaft of metacarpal III is slightly bowed laterally. The shaft is subcircular in section, but it becomes triangular distally. Metacarpal III is slightly shorter than metacarpal II. There is a weak ridge along the ventral surface of the shaft near the distal portion of metacarpal III.

The manual phalanges are relatively long and robust (Fig. 2). The third digit is almost the same length as the second digit. Manual phalanx I-1 is short and powerfully constructed; its diameter is about 19% of its length. The shaft is slightly bowed medially in dorsal view. In ventral view, the shaft is flattened. The shaft is dorsoventrally higher than lateromedially wide. There is a suboval rugosity on the medial surface above the distal end of manual phalanx I-1. The proximal end of manual phalanx I-1 is not well preserved. The ventral surfaces of the proximal and distal ends are slightly concave, and that of shaft is convex.

The first manual ungual is relatively large, strongly curved and has a prominent flexor tubercle. The tubercle extends posteroventrally, and is separated from the ventral margin of the articular end by a distinct groove. The medial surface of the flexor tubercle has a distinct coarse structure. However, its lateral surface is smooth. There is a distinct ridge along the middle of the ventral surface of the flexor tubercle. The ungual is laterally compressed and has a ‘Y’-shaped groove on its medial surface. The lateral surface of the ungual is not exposed, thus its structure remains unclear. There is a proximodorsal lip on the proximal end of the ungual, similar to that of *Machairasaurus leptonychus*^20^, and *Oviraptor philoceratops*^4^, but this lip is reduced in *Citipati osmolskae*^9^, and absent in *Khaan mckennai*^49^.

Manual phalanges II-1 and II-2 are long and moderately robust. Phalanx II-1 is shorter than II-2 and the second ungual. The shafts of II-1 and II-2 are dorsoventrally higher than they are lateromedially wide. In ventral view, the lateral margin of the proximal end expands ventrally, thus forming a distinct concavity between the margins. The central part of the shaft of II-2 is slightly convex ventrally. The collateral ligament pits are shallow concavities. The proximal articular surface of II-2 is highly concave and has an elongate articular heel and tongue. The construction of the articular surface appears to have permitted hyperextension at this joint. The ventral surface of the proximal end of the II-2 is sculptured with short ridges and grooves, and is concave proximally. The collateral ligament pits are deep. The second manual ungual is similar to the first, but slightly smaller, proportionately more elongate, and more weakly curved than the first. The proximodorsal lip is relatively larger, and the flexor tubercle is more strongly developed than in the first ungual. The ‘Y’-shaped groove is also present on the medial surface of the ungual.

Manual phalanges III-1, III-2, and III-3 are relatively short; III-1 and III-2 are subequal in length, and III-3 is longer than the other two. The proximal articular surfaces of III-2 and III-3 are highly concave. As with digit II, the interphalangeal joints appear to have permitted hyperextension of the phalanges. The collateral ligament pits of III-1 and III-2 are shallow concavities, but those of III-3 are deep. The third ungual resembles the ungual of digit II in shape, but is more slender than the first and second unguals. The flexor tubercle of third ungual is also well-developed.

**Figure 5:**
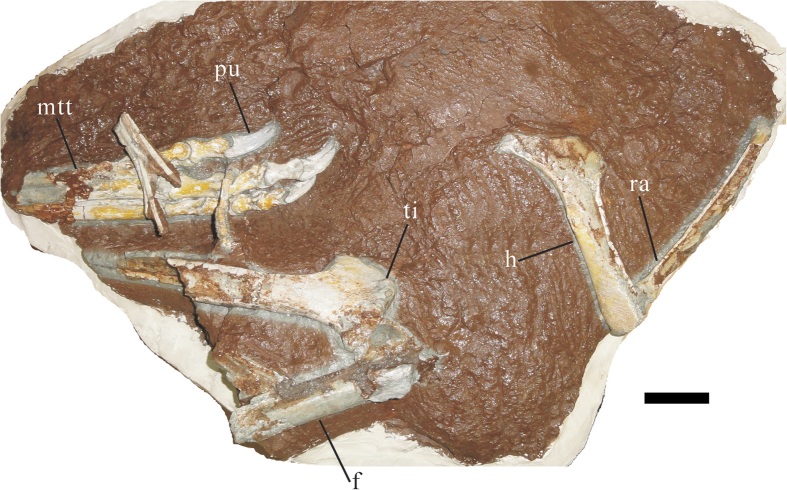
Partial forelimb and hindlimb of *Huanansaurus ganzhouensis* (HGM41HIII-0443) gen. et sp. nov. Abbreviations: f., femur; h., humerus; mtt., metatarsals; pu., pedal ungual; ra., radius; ti., tibia. Scale bar = 5 cm.

The distal portion of the right femur is preserved and it naturally articulated with the proximal end of the right tibia (Fig. 5). The proximal end of the tibia has a distinct cnemial crest. The proximal ends of the metatarsals are missing. Only the dorsal surfaces of the metatarsals are exposed, and the ventral surfaces cannot be observed. Metatarsal III is the most robust and longest of the metatarsals. The distal end of metatarsal III is expanded mediolaterally and is nearly flat on its dorsal surface. The distal end of metatarsal IV is narrow with a convex dorsal surface. The third digit is the longest one. Phalanx III-1 is the stoutest of all. All phalanges have collateral ligament pits. However, the collateral ligament pits on the phalanges of digits III and IV are much deeper than these of the digit II. The pedal unguals are more slender than the manual unguals, and are also weakly curved. The proximal end of each pedal ungual extends strongly posterodorsally. The wide articular ends may have permitted hyperextension of the unguals. The grooves on the medial surfaces of the unguals are more distinct distally than proximally. The ungual of the fourth digit is more curved than the other pedal unguals.

### Phylogenetic analysis of *Huanansaurus*

**Figure 6:**
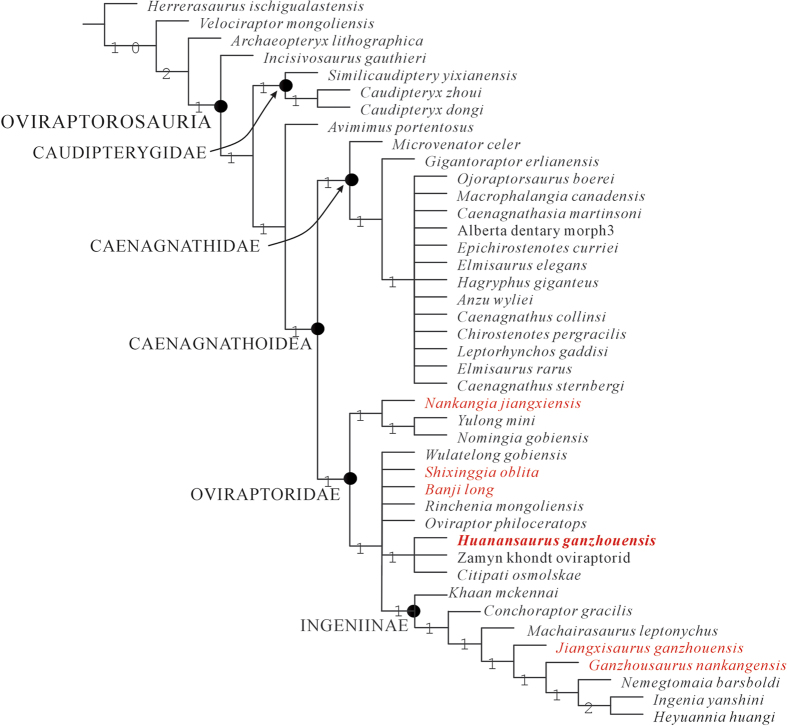
Strict consensus of 60 most parsimonious trees obtained by TNT, based on analysis of 42 taxa and 230 characters, showing the phylogenetic position of *Huanansaurus ganzhouensis* (Tree length = 533, consistency index = 0.510 and retention index = 0.691). Numbers adjacent to each node are Bremer support values Oviraptorids from Nanxiong Formation are in red.

A phylogenetic analysis including 42 taxa (*Herrerasaurus*, *Velociraptor* and *Archaeopteryx* as outgroups; 39 taxa as ingroup) and 230 characters recovers 60 most parsimonious trees. The strict consensus of the most parsimonious trees (Fig. 6) shows that it is almost identical to the tree obtained by Lamanna *et al.*^39^ (see Fig. 6A of Lamanna *et al.*^39^), except for the clade Ingiinae^20^, of which the relationships are better resolved. The strict consensus tree indicates that *Huanansaurus* and other two Mongolian oviraptorids (*Citipati osmolskae* and the Zamyn Khondt oviraptorid) form a polytomy within the clade of Oviraptoridae (Fig. 6), and that they share one synapomorphy: the angle between the ascending and jugal processes of the quadratojugal is less than 90° (character 41, state 1); *Nankangia* appears basal to the clade formed by *Yulong* and *Nomingia*; *Jiangixsaurus* and *Ganzhousaurus* are members of Ingeniinae, the clade proposed by Longrich *et al.*^20^.

The clade Oviraptorosauria is a monophyletic group and is supported by the following synapomorphies: ratio of the preorbital skull length to the basal skull length less than 0.6 (character 1, state 1); the share of the premaxilla (ventral) in the basal skull length 0.12 or more (character 7, state 1); the ratio of the length of the maxilla (in lateral view) to the basal skull length less than 0.4 (character 9, state 1); antorbital fossa bordered anteriorly by the premaxilla (character 14, state 1); basipterygoid processes strongly reduced (character 55, state 1); massive pterygoid–ectopterygoid longitudinal bar present (character 64, state 1); palate extends below the cheek margin (character 65, state 1); extended symphyseal shelf at the mandibular symphysis present (character 74, state 1); mandibular symphysis U-shaped (character 76, state 1); dentary proportionately short and deep, with maximum depth of dentary between 25% and 50% of dentary length (with length measured from the tip of the jaw to the end of the posterodorsal process) (character 78, state 1); ratio of the length of the external mandibular fenestra to total mandibular length 0.25 or more (character 80, state 2); coronoid process anteriorly positioned, near the midpoint of the jaw, with a medially hooked apex (character 88, state 1); splenial strap-like, shallow, not approaching the dorsal mandibular margin (character 94, state 1); mandibular adductor fossa large, anteriorly and dorsally extended, not delimited anteriorly (character 95, state 1) and; dentary teeth absent from tip of jaw but present posteriorly (character 99, state 1).

The clade Caudipterygidae is supported by the following synapomorphies: the ventral ramus of the jugal shallow dorsoventrally or rod-shaped (character 34, state 1); the ascending (squamosal) process of the quadratojugal borders the ventral two thirds or more of the infratemporal fenestra (character 40, state 1); symphyseal portion of the dentary downturned (character 75, state 1); maxillary teeth absent (character 98, state 2); dentary teeth absent (character 99, state 2) and crenulated tomial margin of the premaxilla present (character 161, state 1).

The clade Caenagnathoidea is supported by the following synapomorphies: palatal shelf of maxilla with two longitudinal ridges and tooth-like ventral process (character 11, state 1); pneumatic quadrate (character 45, state 1); dentary short and deep, with maximum depth 50% or more of length (character 78, state 2); mandibular articular facet for quadrate formed exclusively of articular (character 90, state 1); cervical ribs in adults loosely attached to vertebrae (character 104, state 0); lateral pneumatic fossae on the caudal centra present at least in the anterior part of the tail (character 113, state 1); arched iliac dorsal margin (character 136, state 1); mesopubic (i.e., subvertically oriented) pubis (character 145, state 1); pubic shaft concave anteriorly (character 146, state 1); anterior and greater trochanters in contact (character 150, state 1); well-developed adductor fossa and associated anteromedial crest on distal femur (character 153, state 1); ratio of maximum length of metatarsus to that of femur 0.4–0.6 (character 160, state 0); posteroventrally directed retroarticular process (character 198, state 1); and lateral ridge of femur absent or represented by faint rugosity (character 213, state 0).

The Caenagnathidae is supported by the following synapomorphies: preacetabular process expanded ventrally well below the level of dorsal acetabular margin (character 138, state 1); lateral surface of dentary bearing a deep fossa, sometimes with associated pneumatopore (character 167, state 1); and ischial peduncle of pubis with prominent medial fossa (character 201, state 1).

The clade Oviraptoridae is supported by the following synapomorphies: lateral accessory process on the distal end of the quadrate for articulation with the quadratojugal present (character 46, state 1); ratio of external mandibular fenestra height to length 0.7–1.0 (character 79, state 1); anteroventral process of surangular dividing external mandibular fenestra is short and broad (character 81, state 1); dentary posterodorsal ramus strongly bowed dorsally (character 164, state 1); angular largely excluded from external mandibular fenestr by surangular (character 168, state 1); dentary contribution to the external mandibular fenestra relative to the length of dentary greater than 50% (character 171, sate 1); nasal processes of premaxillae anteroposteriorly expanded and mediolaterally compressed to form a bladelike internarial bar (character 183, state 1); and small process of astragalus protrudes through a circular opening at the edge of calcaneum to reach lateral margin of tarsus (character 216, state 1);

The unnamed node (*Nankangia*+(*Yulong*+*Nomingia*)) is supported by a single synapomorphy: posterior haemal arches deeper than long (character 118, state 0).

The clade Oviraptorinae is supported by two synapomorphies: the infratemporal fenestra subquadrate, its anteroposterior length comparable to the orbital length (character 31, state 1), and the deltopectoral crest expanded so it is wider than the shaft diameter (Character 123, state 1).

The clade Ingeniinae is supported by two synapomorphies: dorsal margin of the ilium along the central portion of the blade arched (character 136, state 1) (this character is reversed in *Nemegtomaia*^16^), and ilium low and anteroposteriorly elongate, its height less than 25% of its length (character 180, state 1).

**Figure 7:**
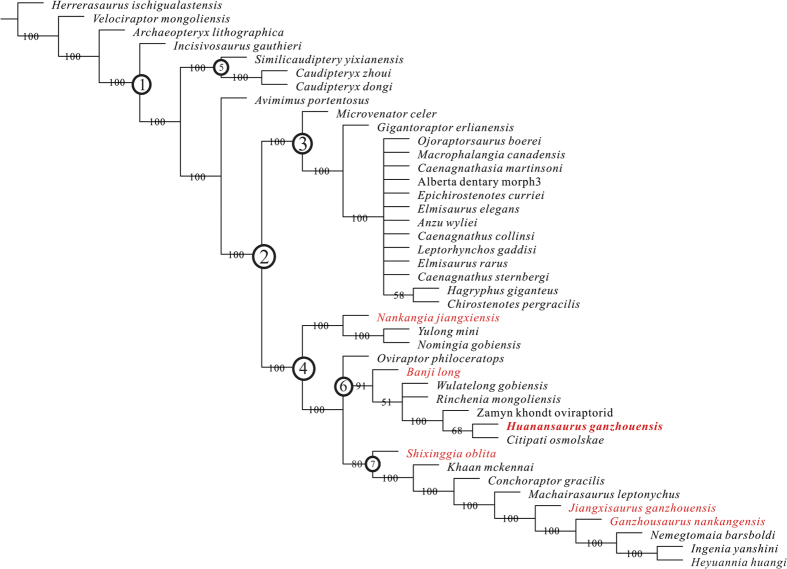
50% majority rule consensus of 60 most parsimonious trees showing better resolved clades of Oviraptorosauria. ① Oviraptorosauria; ② Caenagnathoidea; ③ Caenagnathidae; ④ Oviraptoridae; ⑤ Caudipterygidae; ⑥ Oviraptorinae; ⑦ Ingeniinae. Oviraptorids from Nanxiong Formation are in red.

The 50% majority-rule consensus tree (Fig. 7) shows that Caenagnathidae is better resolved, and that *Hagryphus giganteus* and *Chirostenotes pergracilis* are more closely related to each other than to other caenagnathids. *Nankangia* is basal to the unnamed clade represented by *Yulong* and *Nomingia*. *Banji* is basal to the unnamed clade including taxa such as *Citipati, Huanansaurus, Rinchenia*, *Wulatelong*, and the Zamyn Khondt oviraptorid. Ingeniinae appears to be a monophyletic group that consists of *Conchoraptor gracilis, Khaan mckennai, Heyuannia huangi, Ingenia yanshini, Machairasaurus leptonychus,* and *Nemegtomaia barsboldi*^20^. Because *Ganzhousaurus nankangensis* and *Jiangxisaurus ganzhouensis* occupy positions between *Machairasaurus leptonychus* and *Nemegtomaia barsboldi*, they are recognized here as the members of Ingeniinae. *Shixinggia* is perhaps a basal form within the Ingeniinae, this assessment is congruent with interpretation by Lamanna *et al.*^39^.

## Discussion

*Huanansaurus* is assigned to Oviraptorinae, based on the following characters: the pneumatized crest-like prominence on the skull roof; the anteroventral margin of the premaxilla is inclined anterodorsally relative to the horizontally positioned jugal; the premaxilla projects slightly below the ventrolateral margin of the maxilla; and the postorbital process of the jugal is perpendicular to the ventral ramus of the jugal^15^. However, *Huanansaurus* has some unique characters such as the circular supratemporal fenestra that is much smaller than the lower temporal fenestra, and an external mandibular fenestra that deeply invades the posterodorsal process of the dentary.

Among the oviraptorosaurs with preserved skulls, the cranial morphology of *Huanansaurus* is most similar to that of *Citipati* (Fig. 4), which matches the result of our phylogenetic analysis (Figs 6). The anteroventral corner of the external narial opening is slightly below the horizontal line projected through the posterodorsal corner of the antorbital fenestra (this horizontal line is parallel to the line linking the articular end of the quadrate and the ventral margin of the premaxillae) in *Huanansaurus*, as in *Citipati* and *Yulong* (Fig. 4). The relationship of the position of the posterodorsal corner of the antorbital fenestra and the anteroventral corner of the external narial opening provides additional information on the interrelationships of oviraptorosaurid dinosaurs. The anteroventral corner of the narial opening is much lower than the horizontal line projected through the posterodorsal corner of the antorbital fenestra in *Herrerasaurus*^50^, *Velociraptor*^51^, *Archaeopteryx*^52^ and the primitive oviraptorosaur *Incisivosaurus*, indicating that this is plesiomorphic character, whereas the anteroventral corner of the narial opening is distinctly above the horizontal line in both *Nemegtomaia barsboldi* and *Rinchenia mongoliensis*. The relative positions of the anteroventral corner of the external naris and the posterodorsal corner of the antorbital fenestra in *Anzu*^39^, *Banji*^21^, *Ingenia*^15^, and *Wulatelong*^27^ show that these taxa are more derived than the primitive oviraptorosaurs and less derived than the intermediate forms of *Citipati*^11^, *Huannansaurus*, *Oviraptor*^4^, and *Yulong*^25^ (Fig. 4).

### Paleobiogeographical implications

Southern China (including the Heyuan and the Nanxiong basins of Guangdong Province, and Ganzhou of Jiangxi Province) has become one of the most productive oviraptorosaur localities in the world; the Nanxiong Formation yields perhaps the most diverse oviraptorosaur fauna that has been documented so far^26^.

Oviraptorosaurid dinosaurs discovered in southern China show high taxonomic diversity, and form a distinct faunal assemblage, herein called the Ganzhou Dinosaurian Fauna. Numerous taxa of the oviraptorid dinosaurs discovered in Mongolia come from at least three different formations known as the Djadokhta Formation (*Citipati osmolskae*^10^, *Khaan mckennai*^10^, and *Oviraptor philoceratops*^4^), the Baruungoyot Formation^20^ (*Conchoraptor gracilis*^47^), and the Nemegt Formation^15^: (*Ajancingenia yanshini*^53^ (=*Ingenia yanshini*^44^) and *Rinchenia mongoliensis*^47^); *Nemegtomaia barsboldi*^16^,^17^ comes from both the Nemegt and Baruungoyot formations^22^. There are at least six genera of oviraptorosaurs, including the new taxon, that were recently unearthed from the Nanxiong Formation: *Banji*^21^, *Ganzhousaurus*^28^, *Jiangxisaurus*^29^, *Nankangia*^26^ and *Shixinggia*^18^. Discovery of the new oviraptorid further indicates that the Nanxiong Formation has the most diverse oviraptorosaur fauna compared to all other known localities^26^.

*Huanansaurus* was recovered from the Upper Cretaceous Nanxiong Formation of Ganzhou in Jiangxi Province of China. It is different from any other oviraptorid dinosaur unearthed in southern China. The most closely related to *Huanansaurus* is *Citipati*, which was found approximately 3,000 kilometers northeast in the Djadokhta Formation at Ankylosaur Flats, Ukhaa Tolgod, Gurvan Tes Somon, Omnogov Aimak, Mongolia^10^,^11^. The phylogenetic analysis found that the six oviraptorid taxa from the Nanxiong Formation are nested in three clades of Oviraptoridae (Fig. 6). Their jaw structures are different, the rostral end of the mandibular symphyseal region is not downturned in *Nankangia*^26^, weakly downturned in *Jiangxisaurus*^29^ and *Huanansaurus*, downturned in *Banji*^21^ which may imply they have different foraging strategies, and are likely to occupy different ecological niches of the same biotope. The abundance of oviraptorids from Jiangxi Province suggests that the biotas containing taxonomically and morphologically diversified oviraptorids were more geographically widespread in Asia during the latest Cretaceous, and that the oviraptorids flourished in the Asian terrestrial ecosystems at the end of Mesozoic era.

It should be noted that a recently discovered oviraptorid from central China, *Yulong mini* from the late Late Cretaceous, shares the clade with both the northern *Nomingia* and the southern *Nankangia* – both taxa from Maastrichtian. This allows us to formulate a hypothesis that more than two independent oviraptorid assemblages might be recognized in China during the terminal period of the Cretaceous.

There are, however, some interesting details in speciation between the two assemblages. While at least 12 genera of the northern oviraptorids are known from six formations spanning Campanian to Maastrichtian, the six taxa from the Nanxiong Formation are well constrained temporally and geographically. Moreoveor, southern and central China still remain relatively underexplored regions in comparison with the Gobi Desert. Therefore we assume that all the hypotheses on paleobiogeography of oviraptorids in Asia, formulated or outlined here, will be further critically tested with discovery of new specimens, taxa and sites.

The strict consensus of the most parsimonious trees (Fig. 6) shows that there are repeated sister taxon relationships between the oviraptorosaurs from the Nanxiong Frmation of southern China and those from the Nemegt Basin of Mongolia. It also shows that the North American caenagnathoids form a separate clade, which is exclusive to the oviraptorids of North America. This is consistent with persistent dispersal of oviraptorosaurs in central and eastern Asia in the Late Cretaceous, plus subsequent speciation due to isolation by distance. It appears that representatives of the multiple clades of oviraptorosaurs lived in overlapping geographical areas while being adapted to different niche partitions as well as specialized to various foraging strategies. Because the taxa from the Nanxiong Formation of southern China and the Nemegt Basin of Mongolia are almost randomly distributed across the tree, it implies that the barriers to physical dispersal are low, for example, similar habitats must exist across the geographical area. The barriers to gene flow, however, must be constrained later on due to maximizing distances among ancestral sub-populations led eventually to their isolation and specialization to new evolving paleoenvironmental conditions.

## Methods

In order to determine the phylogenetic position of *Huanansaurus* among Oviraptorosauria, the data matrix of Lamanna *et al.*^39^, which includes most of the published oviraptorosaurs, was used. With the addition of *Huanansaurus* in the modified data matrix of Lamanna *et al.*^39^, 42 taxa (*Herrerasaurus*, *Velociraptor* and *Archaeopteryx* as outgroups; 39 taxa as the ingroup) and 230 characters have been employed. Some scores for *Citipati* and *Jiangxisaurus* in the data matrix of Lamanna *et al.*^39^ were changed based on the publication of a detailed description of *Citipati*^11^ and new observations on *Jiangxisaurus* (Lü, pers. observation) (see Supplementary Information). The phylogenetic analysis was performed using TNT (Tree Analysis Using New Technology) version 1.1 (Willi Hennig Society Edition)^54^. A traditional search (tree bisection-reconnection swapping algorithm, random seeds of 1,000, 1,000 replicates, 10 trees saved per replication) yielded 60 most parsimonious trees of 533 steps, with a consistency index of 0.510 and retention index of 0.691.

### Data archiving

Specimen measurements, the phylogenetic character scores for *Huanansaurus*, and the phylogenetic topology with synapomorphies are available as Supplementary Information.

## Additional Information

**How to cite this article**: Lü, J. *et al.* A New Oviraptorid Dinosaur (Dinosauria: Oviraptorosauria) from the Late Cretaceous of Southern China and Its Paleobiogeographical Implications. *Sci. Rep.* **5**, 11490; doi: 10.1038/srep11490 (2015).

## Supplementary Material

Supplementary Information

## Figures and Tables

**Figure 1:**
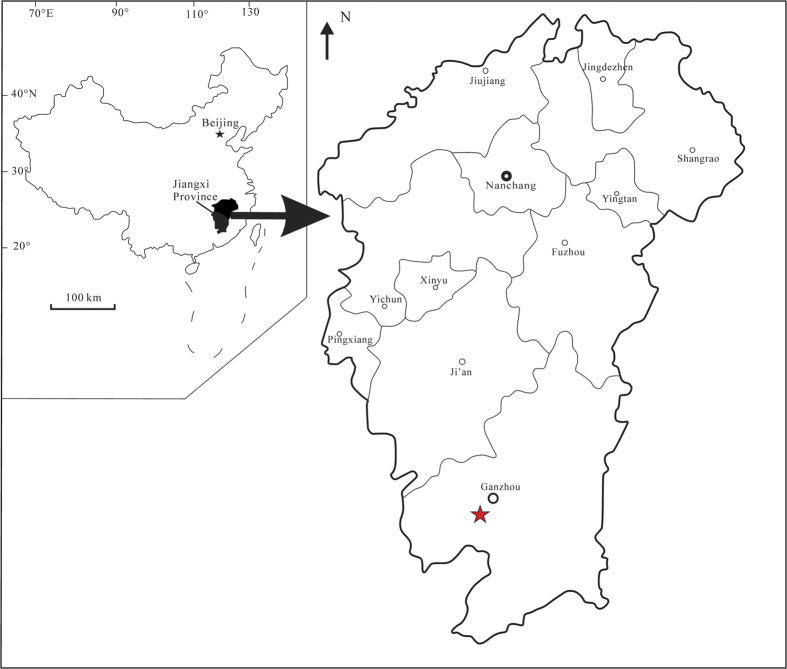
Map of the fossil locality near Ganzhou, Jiangxi Province, southern China. The five-pointed star represents the fossil site. Modified from Lü *et al.*^26^
