# Supplementary material for: A New Oviraptorid Dinosaur (Dinosauria: Oviraptorosauria) from the Late Cretaceous of Southern China and Its Paleobiogeographical Implications
Source: Sci Rep. 2015 Jul 2;5:11490. doi: 10.1038/srep11490 (PMC4489096; doi:10.1038/srep11490)
Supplement: Supplementary Information [file srep11490-s1.docx]

Supplementary Information for:

A New Oviraptorid Dinosaur (Dinosauria: Oviraptorosauria) from the Late Cretaceous of Southern China and Its Paleobiogeographical Implications

Junchang Lü^1*^, Hanyong Pu^2^, Yoshitsugu Kobayashi^3^, Li Xu^2^, Huali Chang^2^, Yuhua Shang^2^, Di Liu^2^, Yuong-Nam Lee^4^, Martin Kundrát^5^and Caizhi Shen^1^

1. Institute of Geology, Chinese Academy of Geological Sciences, Beijing100037, China; Key Lab of Stratigraphy and Paleontology, Ministry of Land and Resources of China, Beijing 100037, China,

2. Henan Geological Museum, Zhengzhou 450016, Henan, China,

3. Hokkaido University Museum, Hokkaido University, Sapporo, Japan,

4. Korea Institute of Geoscience and Mineral Resources, Daejeon, South Korea,

5. Department of Organismal Biology, Evolutionary BiologyCentre, Uppsala University, Uppsala, Sweden.

1. Specimen measurements

2. Photographs of skull and cervical vertebrae of *Huanansaurus ganzhouensis*

3. Phylogenetic analysis

1. **Specimen Measurements**

Table S1. Measurements (in mm) of *Huanansaurus* *ganzhouensis* gen. et sp. nov.

|  | Length | Height (width) |
| --- | --- | --- |
| Skull （from the tip of premaxilla to the posterior margin of parietal） | 199.1 | 101.9 (at the top of the skull (above the orbital) |
| skull (from the tip of premaxilla to the articular end of the quadrate) | 206.1 | 67.55 (at the top of the premaxilla) |
| Preorbital length | 87 | - |
| Maxilla in lateral view | 51 (maximum) |  |
| Basal skull length | 187 | - |
| Orbit | 49.7 | 39.3 |
| Antorbital fenestra | 34.9 | 18.9 |
| Anterior maxillary fenestra | 9.6 | 7.7 |
| Posterior maxillary fenestra | 18.3 | 19.6 (high) |
| Supratemporal fenestra | 26.1 | 26.1 |
| frontal | 29.0 | - |
| Parietal | 47.0 |  |
| nasal | 148.5 (est.) |  |
| Long axis of external nasal naris | 42.3 |  |
| Foramen magnum | 12.3 (width) | 12.7 |
| Lower temporal fenestra | 55.6 | 44.9 |
| Lower jaw | 182.8 | 59.4 |
| Retroarticular process | 20 | - |
| Distance between the vertical level of the anterior margin of the external mandibular fenestra and the coronoid eminence | 40 |  |
| External mandibular fenestra | 24.9 | 31.5 |
| Mandibular symphysis | 34.2 | 38.4 (along the suture) |
| Cervicals 2 to 6 | 230 |  |
|  |  |  |
| humerus | 195 |  |
| radius | 190 |  |
| Metacarpal I, II, III | 40,85,84 | -,11, 9 |
| Manual phalanx I-1,2 | 80,70 |  |
|  |  |  |
| Manual phalanges II-1,2,3 | 55,65,65 |  |
| Manual phalanges III-1, 2, 3, 4 | 32,30,44,52 |  |
| Pedal II-1,2,3 | 45,35,55 |  |
| Pedal III-1,2,3,4 | 45,39,35,50 |  |
| Pedal IV-1,2,3,4,5 | 33,30,21,20,50 |  |

2. Photographs of skull and cervical vertebrae of *Huanansaurus ganzhouensis*


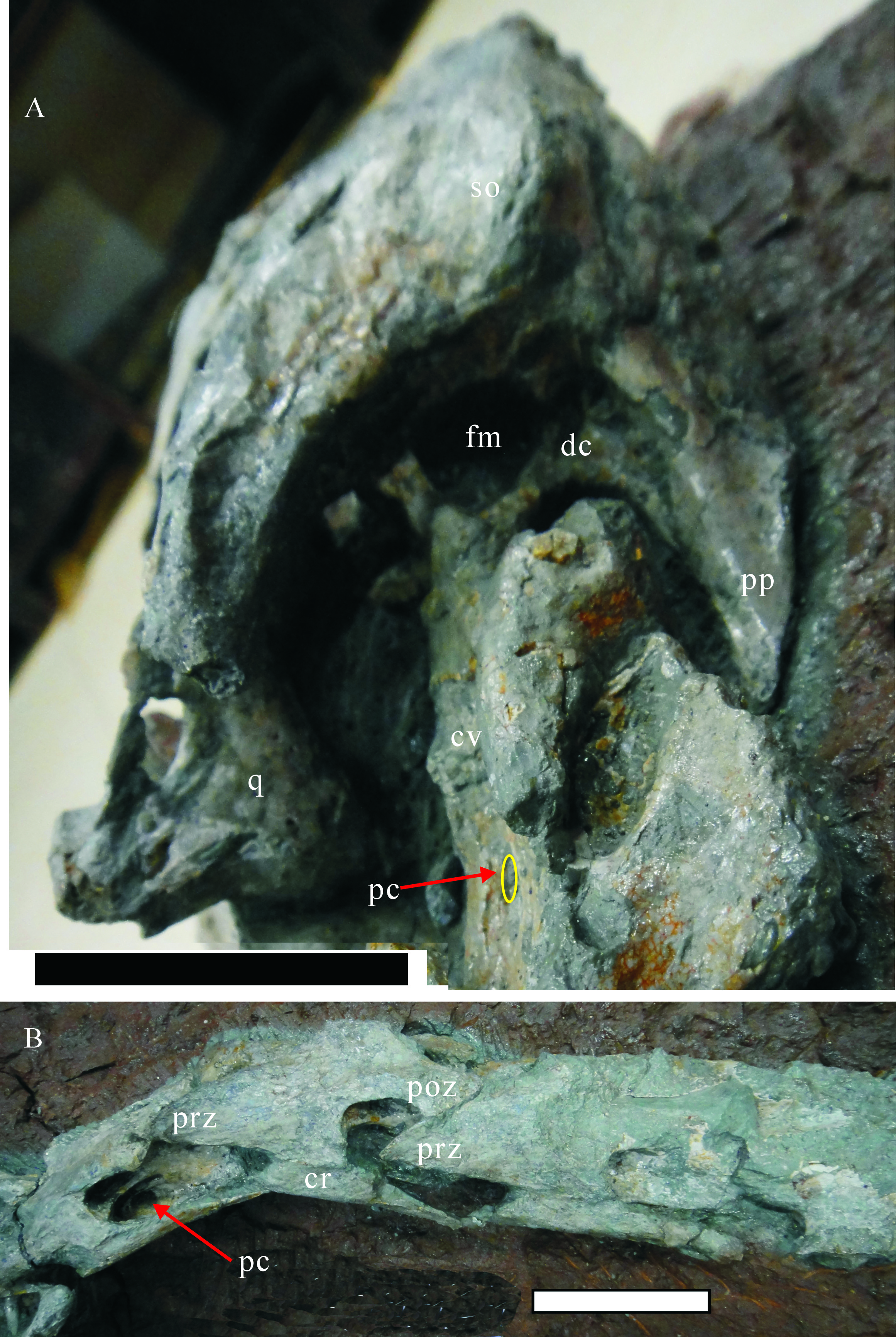


Fig.S1. The skull of Huanansaurus ganzhouensis (HGM41HIII-0443) gen. et sp. nov. in posterior view (A); and the cervical vertebrae in lateral view (B). Abbreviations: cv. cervical vertebrae; cr. cervical rib; dc, distinct concavity; fm. foramen magnum; pc. pleuroceol; pp. paroccipital process; prz. prezygapophysis; poz. postzygapophysis q. quadrate; so. supraoccipital. Scale bar = 2 in A, 5cm in B.

3. Phylogenetic analysis

We included ***Huanansaurus*** in the phylogenetic analysis of **Lamanna et al. ^s1^** (see Methods). The following are the scores **for *Huanansaurus*:**

11222211111101101?11211111111111?1100111111?11100111????1???11?11?????1111111212201100111111????122?110211????????????????001101?211????????????????????????????11011000011?01000??? ?1?1?0????0100000110?????01???0??1??011??101?01???

The other changes to the **Lamanna et al.^s1^** analysis are as follows:

Changed *Citipati osmolskae* from ? to 2 for character 3

Changed *Citipati osmolskae* from ? to 1 for character 8

Changed *Citipati osmolskae* from ? to 0 for character 16

Changed *Citipati osmolskae* from ? to 1 for character 28

Changed *Citipati osmolskae* from ? to 1 for character 32

Changed *Citipati osmolskae* from ? to 1 for character 43

Changed *Citipati osmolskae* from ? to 1 for character 45

Changed *Citipati osmolskae* from ? to 1 for character 48

Changed *Citipati osmolskae* from ? to 1 for character 52

Changed *Citipati osmolskae* from ? to 1 for character 55

Changed *Citipati osmolskae* from ? to 1 for character 58

Changed *Citipati osmolskae* from ? to 2 for character 66

Changed *Citipati osmolskae* from ? to 1 for character 67

Changed *Citipati osmolskae* from ? to 1 for character 69

Changed *Citipati osmolskae* from ? to 1 for character 71

Changed *Citipati osmolskae* from ? to 1 for character 74

Changed *Jiangxisaurus ganzhouensis* from ? to 1 for character 11

Changed *Jiangxisaurus ganzhouensis* from ? to 1 for character 46

Changed *Jiangxisaurus ganzhouensis* from 1 to 2 for character 48

Changed *Jiangxisaurus ganzhouensis* from ? to 1 for character 54

Changed *Jiangxisaurus ganzhouensis* from ? to 2 for character 55

Changed *Jiangxisaurus ganzhouensis* from ? to 0 for character 63

Changed *Jiangxisaurus ganzhouensis* from ? to 1 for character 65

Changed *Jiangxisaurus ganzhouensis* from ? to 2 for character 66

Changed *Jiangxisaurus ganzhouensis* from ? to 1 for character 67

Changed *Jiangxisaurus ganzhouensis* from ? to 0 for character 70

Changed *Jiangxisaurus ganzhouensis* from ? to 1 for character 77

Changed *Jiangxisaurus ganzhouensis* from ? to 2 for character 78

Changed *Jiangxisaurus ganzhouensis* from 0 to 2 for character 80

Changed *Jiangxisaurus ganzhouensis* from ? to 1 for character 82

Changed *Jiangxisaurus ganzhouensis* from ? to 1 for character 84

Changed *Jiangxisaurus ganzhouensis* from ? to 1 for character 89

Changed *Jiangxisaurus ganzhouensis* from ? to 1 for character 106

Changed *Jiangxisaurus ganzhouensis* from ? to 1 for character 107

Changed *Jiangxisaurus ganzhouensis* from ? to 1 for character 110

Changed *Jiangxisaurus ganzhouensis* from ? to 1 for character 119

Changed *Jiangxisaurus ganzhouensis* from ? to 2 for character 120

Changed *Jiangxisaurus ganzhouensis* from 0 to 1 for character 125

Changed *Jiangxisaurus ganzhouensis* from ? to 1 for character 129

Changed *Jiangxisaurus ganzhouensis* from 1 to 2 for character 130

Changed *Jiangxisaurus ganzhouensis* from ? to 1 for character 141

Changed *Jiangxisaurus ganzhouensis* from ? to 1 for character 161

Changed *Jiangxisaurus ganzhouensis* from ? to 1 for character 165

Changed *Jiangxisaurus ganzhouensis* from ? to 0 for character 169

Changed *Jiangxisaurus ganzhouensis* from 0 to 1 for character 171

Changed *Jiangxisaurus ganzhouensis* from ? to 1 for character 172

Changed *Jiangxisaurus ganzhouensis* from ? to 1 for character 184

Changed *Jiangxisaurus ganzhouensis* from ? to 1 for character 185

Changed *Jiangxisaurus ganzhouensis* from ? to 1 for character 186

Changed *Jiangxisaurus ganzhouensis* from ? to 2 for character 189

Changed *Jiangxisaurus ganzhouensis* from ? to 1 for character 192

Changed *Jiangxisaurus ganzhouensis* from ? to 1 for character 198

Changed *Jiangxisaurus ganzhouensis* from ? to 0 for character 200

Changed *Jiangxisaurus ganzhouensis* from ? to 1 for character 207

Changed *Jiangxisaurus ganzhouensis* from ? to 0 for character 217

Changed *Jiangxisaurus ganzhouensis* from ? to 0 for character 218

Changed *Jiangxisaurus ganzhouensis* from ? to 1 for character 219

Changed *Jiangxisaurus ganzhouensis* from ? to 1 for character 220

Changed *Banji long* from 0 to 1 for character 18


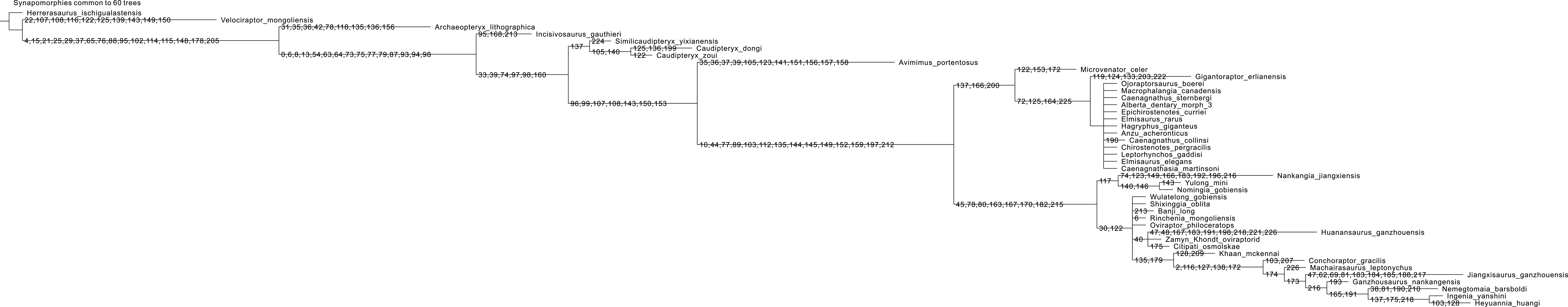


Figure S1. The strict consensus of 60 most parsimonious trees with synapomorphies

References

S1. Lamanna, M.C., Sues, H.D., Schachner, E.R. & Lyson, T.R. A new large-bodied oviraptorosaurian theropod dinosaur from the Latest Cretaceous of Western North America. *PLoS ONE* ***9*,** e92022. doi:10.1371/journal.pone.0092022 (2014).
